# The ReDiKid framework: a transdisciplinary systems approach to child resilience in the digital age

**DOI:** 10.3389/fpsyg.2026.1919590

**Published:** 2026-10-07

**Authors:** Jana Pelclová, Leen d’Haenens, Marc N. Potenza, Jaroslava Suchá, Aleš Gába, Tomas Vetrovsky, David Šmahel, Roman Procházka, Adeela ahmed Shafi

**Affiliations:** 1 Faculty of Physical Culture, Palacký University Olomouc, Olomouc, Czechia; 2 Faculty of Social Sciences, KU Leuven, Leuven, Belgium; 3 Yale School of Medicine, Connecticut Mental Health Center, New Haven, CT, United States; 4 Faculty of Arts, Palacký University Olomouc, Olomouc, Czechia; 5 Faculty of Science, University of Hradec Kralove, Hradec Kralove, Czechia; 6 Faculty of Social Studies, Masaryk University, Brno, Czechia; 7 Centre for Global Wellbeing, University of Gloucestershire, Gloucester, United Kingdom

**Keywords:** 24-h movement behaviors, child resilience, developmental systems, digital adversity, digital environments, digital resilience, self-regulatory capacity, wellbeing

## Abstract

Children’s lives are increasingly shaped by digital environments that influence learning, social relationships, daily routines, and exposure to both opportunities and adversity. However, existing approaches often examine digital experiences, self-regulation, movement behaviors, and social context separately. This paper introduces ReDiKid (Resilient Kid in the Digital World), a developmental-systems framework for understanding resilience in late childhood, with a primary focus on children aged approximately 9–11 years. ReDiKid conceptualizes resilience as a higher-order, adversity-activated process through which adaptive functioning is maintained, restored, or reorganized over time. The framework integrates four interacting domains: psychophysiological, behavioral, contextual, and digital. It proposes four cross-domain pathways, self-regulatory capacity, movement regulation, social buffering, and digital competence, through which resources and vulnerabilities may shape responses to adversity, recovery, and subsequent adaptation. The framework distinguishes digital adversity from normative digital demands and differentiates resilience from competence, adaptive functioning, and multidimensional wellbeing. Six directional and empirically contestable propositions specify expected within-person, between-person, event-level, and contextual patterns, including conditions under which the propositions would not be supported. ReDiKid also recognizes that pathway operation may vary according to developmental stage, sex and gender, structural inequality, cultural context, neurodivergence, disability, and patterns of digital access. To support empirical testing, ReDiKid outlines a multimodal methodological architecture combining intensive longitudinal designs, ecological momentary assessment, wearable sensing, passive digital tracking, contextual data, and advanced longitudinal and multilevel analyses. ReDiKid provides a common conceptual foundation for coordinated study-specific protocols and cumulative theory development concerning child resilience in digitally mediated contexts.

## 1 Introduction

Children’s everyday lives are increasingly shaped by digital technologies that permeate learning, communication, leisure, and sleep (OECD, 2019). Digital environments influence how children acquire knowledge, form social relationships, and structure their daily routines (Livingstone et al., 2018; Odgers and Jensen, 2020). Despite substantial opportunities for learning, creativity, and social connection, digital engagement may also involve challenges, including potentially problematic or functionally impairing patterns of internet use, online social pressure, cyberbullying, exposure to harmful content, physical inactivity, and sleep disturbances (Stiglic and Viner, 2019; Odgers and Jensen, 2020; Smahel et al., 2020). These potentially harmful aspects of digital environments may place additional demands on children’s adaptive and regulatory capacities in everyday life (Orben and Przybylski, 2019; Odgers and Jensen, 2020). The relationship between digital engagement and youth mental health is complex and remains contested. Large-scale studies commonly identify small average associations between digital technology use and wellbeing, while specification-curve analyses demonstrate that estimates may vary according to analytical choices (Orben and Przybylski, 2019; Odgers and Jensen, 2020). At the same time, average associations may obscure substantial heterogeneity between individuals. Intensive longitudinal research indicates that similar forms of social media use may be associated with positive, negative, or negligible changes in momentary wellbeing across different adolescents (Beyens et al., 2020). These findings neither exclude meaningful harms for particular children, activities, or contexts nor establish that digital engagement is uniformly harmful or causally benign. Rather than resolving this broader debate, ReDiKid provides a mechanism-oriented framework for examining when, for whom, and through which psychophysiological, behavioral, contextual, and digital pathways engagement may be associated with adaptive or maladaptive outcomes. Recent policy developments underscore growing societal concern about potential harms and highlight the need for research examining how digital environments shape children’s regulatory processes in everyday life (Australian eSafety Commissioner, 2025; UK Government, 2023).

Resilience, commonly defined as the process of positive adaptation in the context of adversity, has become a central construct for understanding how children navigate complex and changing environments (Masten and Barnes, 2018). Contemporary resilience science emphasizes developmental systems perspectives, highlighting a dynamic interplay between biological, psychological, behavioral, and contextual processes rather than viewing resilience as a stable individual trait (Panter-Brick and Leckman, 2013; Ungar, 2018; ahmed Shafi et al., 2020). From this perspective, resilience is not equated with the absence of psychological symptoms but conceptualized as a functional capacity for regulation, recovery, and adaptation across different contexts.

As children’s everyday environments increasingly include digitally mediated contexts, resilience should also be examined within hybrid offline–online ecologies. Recent research has introduced digital resilience as a domain-specific, risk-activated process referring to young people’s capacity to recognize online risks, mobilize appropriate coping responses, and recover from online adversity (Livingstone et al., 2017; Vissenberg et al., 2022; Pan et al., 2025). However, digital resilience has rarely been integrated into broader developmental systems perspectives that account for children’s psychophysiological regulation, everyday behavioral routines, social contexts, and temporal dynamics.

Recent advances in research on children’s digital lives have generated a large and rapidly growing body of evidence; however, several critical gaps remain. First, existing studies predominantly focus on isolated psychological outcomes or specific risk exposures (Odgers and Jensen, 2020), offering limited insight into how digital experiences interact with children’s everyday regulation across psychophysiological, behavioral, social, contextual, and temporal levels. Second, empirical research has largely concentrated on adolescence (Livingstone et al., 2018), while the transition from childhood to adolescence remains understudied. This period is marked by substantial reorganization of self-regulatory and adaptive capacities, reflected in developments in executive functioning, cognitive flexibility, emotional regulation, and increasing autonomy in everyday decision-making (Best and Miller, 2010; Masten and Barnes, 2018; Zelazo and Carlson, 2012). However, the implications of these developmental changes for resilience in digital contexts remain insufficiently studied. Third, much of the current evidence comes from cross-sectional studies and self-reported data, with limited integration of objective behavioral and psychophysiological measures capable of capturing dynamic regulatory processes as they unfold in real-world contexts. Finally, conceptual work on digital resilience has rarely been translated into comprehensive, staged research programmes that explicitly link theory, empirical testing, and practical implications for education, the prevention of digital harms, mental health promotion, and policy.

This paper introduces the ReDiKid (Resilient Kid in the Digital World) framework, a transdisciplinary conceptual framework that integrates digital ecology with developmental systems theory and a temporal perspective. Here, transdisciplinarity refers to an approach that integrates concepts, methods, and evidence across disciplinary boundaries to develop a shared understanding of complex problems that cannot be adequately addressed within a single disciplinary framework (ahmed Shafi et al., 2023). By providing clearly defined constructs, hypothesized mechanisms, and an integrative conceptual structure, the framework offers a shared reference point for researchers studying child development in digitally mediated environments. It supports the operationalisation of resilience-related variables, informs the design of longitudinal and multimethod studies, and enables more systematic testing of cross-domain pathways linking digital engagement, developmental processes, and adaptive outcomes. Rather than proposing a single fixed model or a protocol for one empirical study, the ReDiKid framework provides a common conceptual foundation for a coordinated programme of complementary studies. Study-specific protocols developed within this programme define their own populations, recruitment strategies, eligibility criteria, timelines, and measurement procedures, while drawing on the shared ReDiKid framework. This structure enables findings from successive studies to be compared, integrated, and used to refine the framework, thereby facilitating theoretically coherent and cumulative research on children’s resilience in the digital age.

ReDiKid contributes to the field in four principal ways. First, it focuses primarily on late childhood, particularly approximately 9–11 years of age, as a context-sensitive developmental period often marked by the transition toward personal ownership of digital devices, increasing susceptibility to peer influence, growing behavioral autonomy, and expanding independent engagement with digital technologies, alongside the continuing maturation of regulatory capacities. This age range represents the primary focus of the framework rather than a universal or sharply bounded developmental window. Second, it embeds digital engagement within a 24-h behavioral ecology, conceptualizing resilience as an embodied and temporally structured process rather than a purely psychological construct. Third, it differentiates between promotive and protective mechanisms across domains, enabling more precise modeling of regulatory functioning under varying levels of adversity. Fourth, it articulates explicit cross-domain pathways and a staged multimodal research architecture, linking theoretical propositions to empirically testable designs.

## 2 Conceptual foundations of the ReDiKid framework

The ReDiKid framework conceptualizes resilience as a dynamic, multilevel process emerging from interactions among individual capacities, behavioral routines, social resources, and environmental conditions over time. It builds on canonical resilience scholarship defining resilience as the maintenance, restoration, or adaptive reorganization of functioning under conditions of significant adversity (Luthar et al., 2000; Masten, 2001, 2021). From a multisystemic perspective, resilience emerges through interactions among biological, psychological, social, institutional, and ecological systems that support adaptation when individuals are challenged by risk or adversity (Ungar and Theron, 2020).

Drawing additionally on Bronfenbrenner’s ecological systems theory (Bronfenbrenner, 1979), ReDiKid assumes that children’s development is embedded within interconnected family, peer, school, community, societal, and historical contexts. Adaptive outcomes are therefore co-constructed across systems rather than residing solely within the individual. This orientation is also consistent with Positive Youth Development (PYD), which emphasizes strengths, agency, supportive relationships, and developmental assets rather than focusing exclusively on risk reduction or deficit prevention (Lerner et al., 2005).

Together, these perspectives provide the foundation for treating resilience as an adversity-activated developmental process while recognizing that many of the same individual and contextual resources may also promote competence and wellbeing in the absence of adversity. Within this ecological perspective, digital technologies should be treated as integral components of children’s developmental environments rather than external stressors or tools. Digital platforms shape cognitive demands, social comparison processes, emotional experiences, and temporal organization of daily life. Whether these influences undermine or support resilience depends on contextual conditions, regulatory capacities, and access to protective resources.

Crucially, resilience is not static. In late childhood, regulatory processes undergo a qualitative shift from predominantly externally scaffolded regulation, provided by parents, teachers, and structured environments, toward more internalized, self-regulatory mechanisms. This period is marked by substantial maturation of executive functions and stress-regulatory systems, reflecting increasing coordination between cognitive control and physiological regulation (Best and Miller, 2010; Blair and Raver, 2015). In parallel, growing integration of cognitive and affective control systems enables more flexible regulation in emotionally salient contexts (Zelazo and Carlson, 2012). Cognitive maturation supports the emergence of reflective thinking, planning, and cognitive reappraisal, while emotional systems become more effectively integrated with executive control functions. Together, these changes reshape how children interpret stressors and how they mobilize coping strategies.

The primary focus on approximately 9–11 years is based on the convergence of several developmental transitions rather than on a single age-specific biological threshold. During late childhood, executive and psychophysiological regulatory systems continue to mature, children become increasingly sensitive to peer evaluation and social belonging, and everyday routines become less exclusively structured by adults. At the same time, late childhood is often a period of rapidly increasing independent access to and ownership of personal digital devices, although the timing and extent of this transition vary substantially across countries, socioeconomic groups, and family contexts. Together, these changes make late childhood a particularly relevant period for examining how emerging regulatory capacities interact with digitally mediated social and behavioral demands.

However, these developmental changes do not occur uniformly or at the same chronological age. Sex and gender, pubertal timing, cognitive and socio-emotional development, family practices, socioeconomic resources, cultural expectations, and patterns of technology access may influence both the timing and the significance of digital experiences. For example, longitudinal evidence suggests that developmental sensitivity to associations between social media use and life satisfaction may emerge at different ages for females and males (Orben et al., 2022). The ReDiKid framework therefore treats chronological age as an approximate developmental marker rather than a sufficient indicator of developmental stage. Empirical applications should, where possible, consider pubertal development, sex and gender, digital autonomy, device access, and relevant cultural and family contexts.

From a developmental systems perspective, resilience during late childhood may be understood as an adversity-activated process emerging through interactions among reflective cognition, emotion regulation, behavioral routines, relational support, and wider ecological conditions. Digital environments may modify these interactions by increasing opportunities for autonomy, learning, social connection, social evaluation, comparison, and exposure to risk. Late childhood therefore represents an important period for examining how adaptive capacities become increasingly self-organized within hybrid offline–online environments.

This position is consistent with multisystemic resilience approaches emphasizing reciprocal interactions among children and their social and ecological environments (Masten, 2021; Ungar and Theron, 2020). It is also compatible with the Dynamic, Interactive Model of Resilience, which highlights the dynamic interaction between individual and system-level processes in educational and learning contexts (ahmed Shafi et al., 2020), and with research on resilient agency in learning (Deakin Crick et al., 2015). Nevertheless, ReDiKid extends beyond an educational-resilience focus by integrating digital ecology, psychophysiological functioning, 24-h movement behaviors, and high-resolution temporal processes within a shared framework.

### 2.1 Relationship to existing frameworks and incremental contribution

ReDiKid builds on several complementary theoretical traditions but is not reducible to any single existing framework. Ecological systems approaches explain development through reciprocal interactions between the child and interconnected environmental systems operating across multiple levels and over time. Positive Youth Development approaches emphasize individual strengths, developmental assets, agency, supportive relationships, and opportunities for thriving and constructive engagement with the wider community (Benson et al., 2006; Lerner et al., 2005). ReDiKid retains this strengths- and assets-based perspective while extending it by explicitly examining how individual and contextual resources function promotively and protectively when children encounter adversity within digitally embedded developmental environments.

Resilience frameworks, including multisystemic approaches and the Dynamic, Interactive Model of Resilience (DIMoR), explain how adaptation emerges through dynamic interactions between individuals and their environments under challenging conditions. Media-effects frameworks such as the Differential Susceptibility to Media Effects Model (DSMM) explain why media effects vary according to dispositional, developmental, and social susceptibility and through media-response states. Research on information and communication technologies (ICTs) is further informed by the Integrative Model of ICT Effects on Adolescents’ Well-being (iMEW), which integrates developmental, media, communication, and health perspectives to explain associations between ICT use and adolescent wellbeing. By contrast, the Interaction of Person–Affect–Cognition–Execution model (I-PACE) focuses primarily on mechanisms involved in the development and maintenance of addictive and other maladaptive behaviors.

ReDiKid draws selectively on these contributions rather than proposing an alternative to them. Its distinctive purpose is to explain resilience processes in digitally embedded late childhood by integrating an explicit adversity criterion with psychophysiological functioning, 24-h movement behaviors, relational and structural contexts, and the qualitative and technological characteristics of digital engagement. It further specifies how these processes may be examined across event-level, day-level, and developmental timescales using complementary multimodal methods. Table 1 summarizes the dimensions of the existing frameworks that are most directly relevant to the incremental contribution of ReDiKid.

**TABLE 1:** Comparison of ReDiKid with related developmental, resilience, and digital-media frameworks.

Framework	Main emphasis	Adversity and temporal focus	What ReDiKid retains and adds
Bronfenbrenner’s ecological systems theory (Bronfenbrenner, 1979)	Development through reciprocal interactions between the child and nested ecological systems, ranging from proximal relationships to broader societal contexts and changing over time.	Adversity is not required. Primarily developmental and contextual, with change understood across interacting ecological systems and developmental time.	ReDiKid retains the ecological view of development as embedded within interconnected systems. It extends this perspective by explicitly differentiating the digital environment as a developmental domain and by specifying adversity-activated resilience processes within digital and 24-hour behavioral contexts.
PYD (Benson et al., 2006; Lerner et al., 2005)	Positive development through individual strengths, developmental assets, agency, supportive relationships, and the 5Cs, contributing to thriving and engagement with the wider community.	Adversity is not required. Primarily focuses on positive developmental trajectories and the accumulation of strengths and developmental assets over time.	ReDiKid retains the strengths- and assets-based emphasis on individual and contextual resources. It extends PYD by examining how these resources operate promotively and protectively when adversity occurs, including how challenging or negative experiences may, under supportive conditions, contribute to recovery, adaptation, and resilience development in digitally embedded childhood.
DIMoR (ahmed Shafi et al., 2020)	Dynamic and relational resilience processes in education and learning contexts.	Challenge and adversity are central; broad developmental and systemic perspective.	ReDiKid retains the dynamic and relational understanding of resilience. It extends this perspective to digitally embedded late childhood by integrating digital ecology, psychophysiological functioning, 24-h movement behaviors, and multimodal measurement across event-, day-, and developmental timescales.
DSMM (Valkenburg and Peter, 2013)	Individual differences in susceptibility to media effects through dispositional, developmental, and social factors.	Adversity is not required; includes short- and longer-term media effects.	ReDiKid incorporates differential susceptibility but treats it as one component of a broader resilience process. It focuses specifically on how susceptibility shapes exposure, regulation, recovery, and adaptation when digital experiences become stressful, harmful, or otherwise adverse.
iMEW (Smahel et al., 2023)	Integration of developmental, media, behavioral, and health perspectives on ICT use and adolescent wellbeing.	No explicit adversity criterion; primarily developmental and longitudinal.	ReDiKid retains the integration of media, development, behavior, and health. It adds a late-childhood focus, an adversity-activated process model, event- and day-level dynamics, 24-h movement behaviors, and psychophysiological and sensor-based measurement.
I-PACE (Brand et al., 2019)	Development and maintenance of problematic or addictive behaviors through person, affective, cognitive, and executive processes.	Focuses on progression towards maladaptive behavior rather than adversity-defined resilience.	ReDiKid retains attention to affective, cognitive, and executive regulation. It broadens the outcome space beyond pathology by examining adaptive, neutral, and harmful digital engagement, as well as the resources and processes that enable recovery after adverse experiences.
ReDiKid (present framework)	Resilience in digitally embedded late childhood across psychophysiological, behavioral, contextual, and digital domains.	Explicit acute or chronic adversity criterion; event-level, day-to-day, and developmental processes.	ReDiKid brings together adversity-activated resilience, developmental susceptibility in late childhood, digital ecology, 24-h movement behaviors, multimodal measurement, and directional propositions about how processes unfold across domains and timescales within a shared conceptual architecture.

The contribution of ReDiKid therefore lies not in introducing entirely new constructs, but in organizing established resilience, developmental, behavioral, and media-effects concepts within a common, adversity-activated and empirically testable framework. Its specific contribution is the integration of psychophysiological regulation, 24-h movement behaviors, contextual resources, and qualitative and technological features of digital engagement across multiple temporal scales.

## 3 The ReDiKid framework

Figure 1 presents the overall structure of the ReDiKid framework, including the four domains, their interconnections, and their temporal embeddedness. The framework comprises four interrelated domains that are analytically distinct yet dynamically interconnected. The psychophysiological and behavioral domains primarily capture embodied regulatory processes, whereas the contextual and digital domains capture the broader environments in which these processes unfold. More specifically, the psychophysiological domain captures children’s regulatory responses and capacities; the behavioral domain captures the timing and organization of daily activities across the day; the contextual domain captures relational and institutional structures; and the digital domain captures the content, purpose, social meaning, and technological conditions of digitally mediated engagement.

**FIGURE 1:**
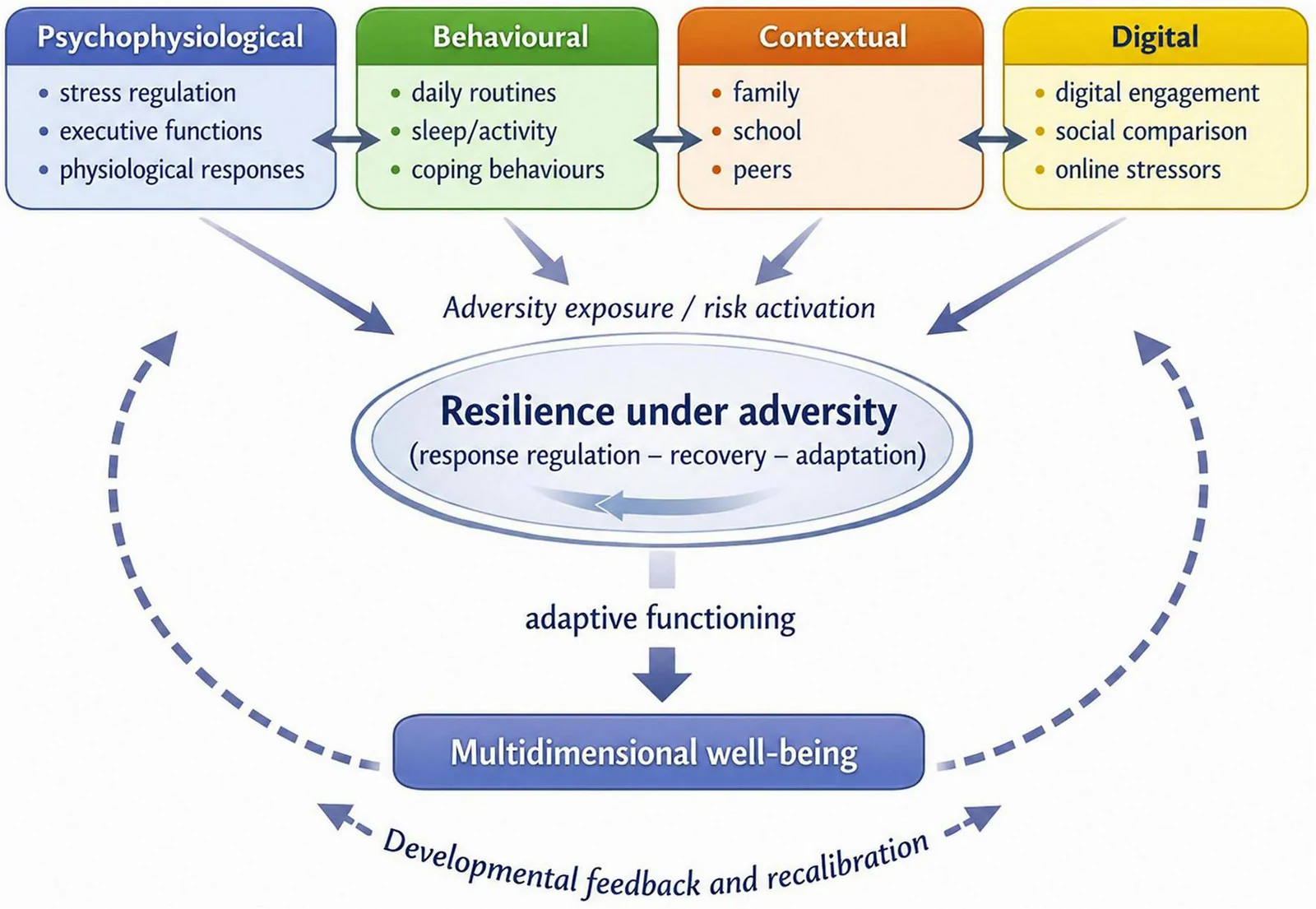
The ReDiKid framework: a dynamic developmental-systems representation of child resilience under adversity and multidimensional wellbeing. Resilience is activated when demands or exposures meet the specified adversity criterion. In the absence of adversity, the same individual and contextual resources may support competence, adaptive functioning, and wellbeing without constituting a resilience process.

Although these domains are analytically distinct, they are not mutually exclusive. The same experience may involve more than one domain, depending on the level of analysis. The digital domain, in particular, cuts across and interacts with the other three: digitally mediated experiences may affect children’s psychophysiological regulation, shape behavioral routines and temporal patterns, and be embedded in wider relational, family, school, and institutional contexts.

Within the framework, resilience is understood as emerging from continuous interactions among these domains over time. Resilience is conceptualized as a higher-order, adversity-activated process inferred from the maintenance, restoration, or adaptive reorganization of functioning over time rather than as a regulatory capacity residing within the child. Their influences converge on a cyclical process of response regulation, recovery, and adaptation. Response regulation refers to the initial cognitive, emotional, behavioral, and physiological adjustments that occur during or following adversity. It is therefore one phase of the resilience process and should be distinguished from self-regulatory capacity, which represents an antecedent capacity that may shape the magnitude of reactivity and the speed or completeness of recovery. This resilience process is iterative and context-sensitive, reflecting ongoing adjustments in regulatory responses across time and situations. It emerges through interactions between children’s embodied regulatory capacities and the broader developmental ecologies in which they are embedded. The framework therefore adopts a temporal perspective, emphasizing how cognitive, emotional, behavioral, and physiological demands are coordinated across daily life.

Consistent with the framework’s systems-oriented perspective, resilience is activated under conditions of adversity and refers to the processes through which adaptive functioning is maintained, restored, or reorganized. Adaptive functioning is conceptualized as a proximal and context-specific indication of the child’s capacity to meet age-relevant developmental demands during or following adversity, whereas multidimensional wellbeing represents a broader developmental outcome that may emerge over time. In the absence of specified adversity, positive functioning should be described as competence, adaptive functioning, or wellbeing rather than as resilience (Luthar et al., 2000; Masten, 2001).

Following adversity, adaptive outcomes may involve the maintenance or restoration of previous functioning or the development of new patterns of adaptation and resilient reintegration (Richardson, 2002; Vella and Pai, 2019; Waugh and Sali, 2023). The framework therefore incorporates a developmental feedback and recalibration loop, indicating that previous experiences and outcomes influence subsequent configurations of regulatory capacities and environmental conditions. These feedback processes may shape future resilience trajectories. Resilience is activated when demands or exposures meet the specified adversity criterion. In the absence of adversity, the same individual and contextual resources may support competence, adaptive functioning, and wellbeing without constituting a resilience process.

Figure 1 provides a common conceptual architecture for developing study-specific empirical models. ReDiKid is not intended as a single fixed statistical model, and individual studies may operationalise selected domains, constructs, and pathways according to their research questions and methodological designs. However, this flexibility does not imply that any empirical pattern is consistent with the framework. Each empirical application should specify a priori the relevant adversity or exposure condition, the proposed mechanism, the level of analysis, the temporal ordering of the constructs, and the expected direction of association. Findings that do not demonstrate the predicted direction, temporal sequence, or moderating pattern under the specified conditions should be interpreted as potentially challenging the corresponding proposition and should inform its subsequent refinement, delimitation, or, where evidence accumulates, rejection. In this way, ReDiKid supports methodological diversity while remaining empirically contestable and capable of cumulative theory development.

The framework integrates resilience theory with systems-oriented and temporal perspectives, providing a conceptual foundation for staged empirical research. Across all four domains, it distinguishes analytically between promotive and protective mechanisms. Promotive factors are conceptualized as resources or conditions that support adaptive functioning across varying levels of adversity, whereas protective factors are conceptualized as mechanisms that may reduce, buffer, or alter the impact of elevated stress or digitally mediated adversity. These categories are intended to guide theory-driven model development rather than prescribe a fixed analytic role for each variable. Depending on the research question, causal assumptions, and methodological design, the same variable may be specified as a predictor, covariate, mediator, moderator, or outcome-relevant mechanism. For example, stable behavioral rhythms may support baseline regulatory functioning, whereas parental mediation, peer support, or teacher support may buffer the regulatory demands associated with online exclusion or cybervictimisation. Within school contexts, teachers may serve as important protective resources by fostering inclusion, responding to peer conflict, and supporting children in navigating challenging digital experiences.

### 3.1 Psychophysiological domain

The psychophysiological domain captures regulatory processes that support adaptation, including emotional regulation, cognitive flexibility, stress responsivity, and perceived regulatory efficacy. Within the construct hierarchy of ReDiKid, self-regulatory capacity represents a broad antecedent capacity encompassing the goal-directed regulation of cognitive, emotional, behavioral, and physiological responses. Emotion regulation is conceptualized more narrowly as a specific component of self-regulation concerned with influencing the trajectory, experience, or expression of emotional responses. Consistent with Gross (2015) extended process model, emotion regulation may involve identifying the need to regulate, selecting an appropriate strategy, and implementing that strategy.

Executive functions, including inhibitory control, working memory, and cognitive flexibility, represent cognitive capacities that support self-regulation but should not be treated as equivalent to it. Effortful control is similarly related but refers primarily to a temperament-based disposition towards voluntary attentional and behavioral control. Psychophysiological indicators are therefore treated as antecedent capacities or response mechanisms rather than as resilience outcomes in themselves. They inform how children respond to and recover from cognitive, emotional, sensory, social, and executive demands in offline and digital contexts.

During late childhood, these systems undergo important developmental recalibration. Increasing integration between prefrontal regulatory networks and autonomic stress systems supports a gradual shift from predominantly reactive stress responses toward more flexible and context-sensitive regulation. This period is characterized by improving autonomic flexibility and more efficient recovery following stress exposure, reflecting the maturation of regulatory control (Gunnar and Quevedo, 2007; Blair and Raver, 2015). Improvements in executive functions such as inhibitory control and cognitive flexibility may further strengthen the regulation of stress responses (Best and Miller, 2010).

Importantly, stress responsivity is not conceptualized as a fixed vulnerability marker but as a system shaped by environmental demands and developmental history (Del Giudice et al., 2011). From this perspective, variability in stress reactivity reflects context-sensitive calibration rather than simple dysregulation.

Indicators such as autonomic flexibility, stress responsivity, and recovery dynamics therefore provide insight not only into physiological functioning but also into the organization of regulatory capacity. In the context of increasing digital cognitive load and social evaluative exposure, these developing capacities may become particularly relevant.

### 3.2 Behavioral domain

Behavioral routines constitute a key domain through which resilience processes are enacted. The behavioral domain conceptualizes physical activity, sedentary behavior, and sleep as interrelated behaviors across the 24-h day that support recovery, regulation, and adaptation (Tremblay et al., 2016; Pedišić et al., 2017). Behavioral patterns are conceptualized not merely as lifestyle indicators but as mechanisms through which resilience may be maintained, strengthened, or depleted across daily cycles.

Late childhood represents a developmental period in which behavioral regulation becomes increasingly self-directed. During late childhood, and with considerable variation across children and contexts, children typically gain greater autonomy over physical activity, sedentary behavior, digital engagement, sleep timing, and peer-related activities. This shift coincides with growing executive capacity and expanding peer orientation, making daily routines increasingly reflective of emerging self-regulatory competencies rather than exclusively externally structured schedules (Best and Miller, 2010).

Early shifts toward later chronotypes and increasing exposure to evening digital engagement may contribute to irregular sleep timing, reduced sleep regularity, and insufficient sleep duration and quality, placing additional demands on regulatory systems. Behavioral regulation during this period therefore reflects an evolving balance between emerging autonomy and the structuring influence of environmental contexts.

Within the ReDiKid framework, digital screen engagement is considered part of the 24-h behavioral continuum when examined in terms of timing, duration, and temporal distribution. However, the content, meaning, cognitive demands, and social context of digitally mediated interaction are addressed separately within the digital domain.

### 3.3 Contextual domain

The contextual domain encompasses the relational, institutional, and environmental systems that scaffold or constrain regulatory development. It includes family support, peer relationships, classroom climate, school structures, and broader socio-environmental conditions. These contextual resources are conceptualized not as static background variables but as dynamic systems that actively shape children’s access to adaptive opportunities and may function both promotively and protectively (Masten and Barnes, 2018; ahmed Shafi et al., 2020). Promotively, they provide stable relational and structural scaffolding that supports adaptive functioning across everyday contexts; protectively, they may buffer the impact of socially evaluative stressors or digital adversity by offering compensatory support.

The contextual domain also includes structural conditions that influence children’s access to digital opportunities, their exposure to risk, and their capacity to mobilize support. These conditions include socioeconomic resources, the availability and quality of connectivity and devices, opportunities to develop digital skills, language and migration-related circumstances, disability and neurodivergence, gendered expectations, discrimination, and unequal access to family, school, community, and professional support. Such factors should not be treated merely as background covariates because they may alter the likelihood, meaning, and consequences of digital adversity and the operation of the proposed pathways.

Structural disadvantage does not imply uniform vulnerability, nor do disability or neurodivergence necessarily predict poorer digital outcomes. Digital environments may also provide opportunities for communication, learning, identity exploration, social participation, and access to support that are less readily available in offline settings. Vulnerability and protection are therefore understood as relational and context-dependent, emerging through interactions among children’s characteristics, available resources, social and institutional responses, and the accessibility and design of digital environments (Hassrick et al., 2021; UNICEF Innocenti, 2025).

From a developmental perspective, late childhood represents a period of significant reorganization within this domain. While parental support remains a primary anchor of emotional security and regulatory scaffolding, peer relationships gain increasing salience for self-evaluation, competence beliefs, and emerging social identity (Eccles, 1999). Children become more sensitive to social comparison, feedback visibility, and belongingness, with these developmental sensitivities increasingly expressed across both face-to-face and digitally mediated environments.

As peer interaction becomes increasingly embedded in online communication platforms, contextual influences extend beyond physical settings into continuously accessible digital spaces. Digital communication may intensify visibility, social comparison, and evaluative exposure, thereby amplifying the regulatory significance of peer-related experiences (Nesi et al., 2018). However, the underlying relational and institutional structures, such as family, peers, and school, remain conceptually distinct from the digital platforms and communication channels through which they are expressed.

Institutional structures, including school policies, organizational practices, and the availability of extracurricular opportunities, represent structural contexts that shape opportunities for behavioral regulation, mastery experiences, and social integration. These environments provide predictable routines, social feedback, and exposure to challenge, thereby functioning as developmental scaffolds that interact with children’s emerging self-regulatory capacities.

Contextual resources are operationalised through children’s perceived access to relational and institutional support, reflecting the extent to which their environments provide conditions conducive to coping, recovery, and adaptive growth.

In addition to child-perceived indicators of support, family-level processes may be assessed through parallel caregiver reports collected alongside child measures. This linked parent–child design allows contextual scaffolding, digital household practices, and regulatory structures to be examined beyond child self-report alone, strengthening the ecological validity of the contextual domain.

### 3.4 Digital domain

Whereas the contextual domain captures relational and institutional structures, the digital domain refers to the technological environments (typically digital media) and digitally mediated interaction within which regulatory processes unfold. The digital domain addresses the dual role of digital technologies as both developmental resources and potential sources of harmful content, social pressure, or stress (Christakis and Hale, 2025). Digital engagement is conceptualized in relation to content, purpose, social context, and individual susceptibility, rather than as a function of duration alone.

Within the ReDiKid framework, digital environments are conceptualized as hybrid social spaces in which regulatory skills are continuously exercised. Parental mediation, digital literacy, and structural access conditions are understood as contextual factors that shape how digital experiences interact with emerging self-regulatory capacities. Digital engagement is therefore treated neither as inherently harmful nor as inherently beneficial, but as developmentally embedded and differentially experienced.

Within this domain, digital resilience is conceptualized as a domain-specific manifestation of the broader resilience process when the activating adversity is digitally mediated, rather than as a separate or parallel form of resilience. For empirical purposes, it may be operationalised as a multidimensional latent construct capturing children’s appraisal of online risks, mobilization of adaptive coping responses, response regulation, and recovery following digital adversity (Vissenberg et al., 2022; Pan et al., 2025). It is therefore conceptually distinct from antecedent capacities such as digital literacy and digital competence, while following the same defining logic as resilience more broadly: adaptive functioning must be maintained, restored, or adaptively reorganized under conditions of adversity. Positive or skilled digital functioning in the absence of adversity reflects digital competence or wellbeing rather than digital resilience.

Rather than focusing solely on screen time or on broad distinctions between “active” and “passive” social media use, the ReDiKid framework acknowledges that the effects of digital engagement depend on developmental, relational, dispositional, social, and cultural factors (Valkenburg, 2020; Smahel et al., 2023; Godard and Holtzman, 2024). This perspective recognizes that categories such as active and passive use may be difficult to separate in contemporary social media environments and that their implications for wellbeing depend on the context, purpose, and meaning of engagement (Verduyn et al., 2017; Godard and Holtzman, 2024).

During late childhood, increasing peer salience and sensitivity to social comparison may coincide with more autonomous engagement in digitally mediated communication. The timing and intensity of these changes vary according to pubertal development, sex and gender, family and cultural context, and children’s access to digital technologies. As children increasingly engage with digitally mediated communication, online interactions become intertwined with evolving self-concept and perceived social standing. In this period, digital environments may exert heightened regulatory demands, particularly in contexts involving visible feedback, peer evaluation, or social exclusion (Eccles, 1999; Nesi et al., 2018).

#### 3.4.1 Digital adversity and risk activation

Within the ReDiKid framework, digital adversity is defined as a digitally mediated event or exposure whose severity, persistence, or functional significance is sufficient to threaten or meaningfully disrupt age- and context-appropriate functioning. Digital adversity therefore involves more than the presence of discomfort, challenge, social evaluation, or temporary negative affect. Its identification requires consideration of the nature and severity of the experience, its duration or recurrence, the child’s perceived threat or loss, its interference with everyday functioning, and the developmental, relational, and cultural context in which it occurs (Luthar et al., 2000; Masten, 2001).

The framework does not assume a single universal numerical threshold separating adversity from normative demands. Instead, empirical studies should define adversity criteria a priori and specify the indicators through which severity, persistence, and functional impact will be assessed. Table 2 distinguishes acute or event-based digital adversity, chronic or exposure-based digital adversity, and normative developmental demands.

**TABLE 2:** Taxonomy and classification criteria for digital adversity within the ReDiKid framework.

Category	Operational definition	Illustrative examples	Classification criterion
Acute or event-based digital adversity	A discrete and identifiable digitally mediated event with sufficient severity or perceived threat to disrupt, or credibly threaten, age-appropriate emotional, behavioral, social, academic, or physiological functioning.	Cybervictimisation; severe online exclusion or humiliation; coercion or manipulation; non-consensual sharing of personal content; unwanted exposure to seriously harmful content.	The event should involve meaningful threat, harm, loss, humiliation, or functional disruption beyond an ordinary transient challenge.
Chronic or exposure-based digital adversity	Repeated, persistent, or cumulative digital exposure that creates sustained regulatory burden or progressively interferes with everyday functioning.	Persistent online harassment or exclusion; repeated exposure to harmful content; sustained evaluative visibility; recurrent algorithmically amplified social or commercial pressures; repeated digitally related disruption of sleep or daily routines.	Classification should consider duration, recurrence, cumulative burden, reduced opportunity for recovery, and evidence of sustained functional impact.
Normative developmental demands	Common digital experiences that may require adjustment or self-regulation but do not, by themselves, constitute significant adversity.	Ordinary social comparison; visible feedback; occasional disagreement; learning to navigate a new platform; minor disappointment; everyday management of notifications or digital demands.	These experiences should not be classified as adversity unless their severity, repetition, perceived threat, or functional impact crosses a prespecified study-specific threshold.

These categories are analytically distinct but may overlap over time. A discrete event may become chronic when it is repeated, continuously visible, or amplified through digital networks, while an initially normative demand may become adversity when its intensity, persistence, or functional impact exceeds the defined threshold. Conversely, exposure to a digital risk should not automatically be equated with experienced adversity, because its meaning and impact may vary according to the child’s developmental capacities, prior experiences, available support, and broader social and cultural context.

This distinction is particularly important for identity-related digital experiences. Social comparison, feedback, experimentation with self-presentation, and exploration of social identity may constitute normative developmental processes and may have supportive, neutral, or challenging functions. They should therefore not be treated as adversity solely because they occur in digital environments (Granic et al., 2020).

Digital adversity serves as the activating condition for digital resilience. Digital competence refers to the knowledge, critical understanding, and skills that support effective engagement with digital environments, whereas digital resilience refers specifically to regulatory, coping, and recovery processes expressed under conditions of digital adversity. Adaptive functioning is the proximal indication that age-relevant functioning has been maintained, restored, or reorganized, while multidimensional wellbeing represents a broader developmental outcome. Thus, positive digital functioning in the absence of adversity reflects competence or wellbeing rather than resilience.

#### 3.4.2 Adaptive, compensatory, excessive, and harmful digital engagement

Digital engagement should not be classified as adaptive or maladaptive solely on the basis of its duration or frequency. Its developmental significance depends on the function of the engagement, the context in which it occurs, the child’s motivations and needs, the flexibility of the behavior, and its short- and longer-term consequences. The Compensatory Internet Use Model proposes that digital engagement may serve as a response to psychosocial difficulties or unmet offline needs, for example by providing temporary relief from distress, opportunities for social connection, or access to experiences that are less readily available offline (Kardefelt-Winther, 2014). Compensatory use is therefore not inherently pathological. It may remain adaptive or neutral, but it may become maladaptive when it becomes persistent and inflexible, reinforces avoidance, substantially displaces sleep, physical activity, relationships, or school participation, or contributes to meaningful distress or functional impairment.

Within ReDiKid, adaptive digital engagement refers to flexible and context-appropriate participation that supports learning, communication, recreation, identity development, or social connection without meaningful impairment. Compensatory engagement refers to the use of digital environments to regulate distress or address unmet psychological or social needs and may have adaptive, neutral, or harmful consequences. Excessive engagement refers descriptively to a high duration or frequency of use but is not, by itself, evidence of problematic or compulsive behavior. Harmful or problematic engagement refers to a persistent pattern associated with significant distress, impaired functioning, reduced behavioral flexibility, or adverse developmental consequences. These patterns are not necessarily fixed categories and may change over time as children’s needs, resources, contexts, and patterns of use change.

The relationship between the amount of digital engagement and wellbeing may also be non-linear. The Goldilocks Hypothesis suggests that moderate digital engagement is not necessarily harmful and that associations may differ at higher levels of exposure (Przybylski and Weinstein, 2017). However, ReDiKid does not assume a universal quantitative threshold separating beneficial from harmful use. Such thresholds may vary according to the type and purpose of the activity, its timing and content, the child’s developmental characteristics, the outcome being considered, and the wider family and cultural context.

To avoid over-pathologising children’s everyday digital lives, high engagement, strong interest, or the use of digital media for coping should not automatically be interpreted as evidence of addiction or disorder. Terms such as problematic, compulsive, or addictive use should be reserved for patterns characterized by persistence, impaired control or flexibility, clinically or developmentally meaningful distress, and interference with important areas of functioning (Billieux et al., 2015). ReDiKid therefore considers both the regulatory benefits and the potential costs of digital engagement and examines the conditions under which initially adaptive or compensatory use may become harmful.

### 3.5 Construct hierarchy and discriminant definitions

To avoid conceptual overlap, the ReDiKid framework distinguishes five functional levels: (1) adversity as an activating condition; (2) antecedent resources and vulnerabilities; (3) response mechanisms; (4) resilience as a higher-order temporal process; and (5) proximal and distal developmental outcomes. Constructs may be associated across these levels, but they should not be treated as interchangeable. In particular, capacities that support the management of adversity are not themselves evidence of resilience unless adaptive functioning is maintained, restored, or reorganized under specified adversity conditions. Table 3 summarizes the definitions and discriminant criteria for the central constructs.

**TABLE 3:** Construct hierarchy and discriminant definitions within the ReDiKid framework.

Construct	Definition and conceptual role	Primary level	Illustrative operationalisation	Discriminant criterion
Adaptive functioning	Proximal, context-specific capacity to meet age-relevant developmental demands during or following adversity.	Event, daily or medium-term outcome.	Emotional stability, school participation, social functioning, routine maintenance.	A proximal outcome through which resilience is evaluated, not the process itself.
Digital adversity	Activating event or exposure whose severity, persistence, or functional significance threatens or disrupts age-appropriate functioning.	Event, exposure, day or longer-term context.	Cybervictimisation, persistent exclusion, harmful-content exposure, cumulative digital pressure.	Unlike ordinary digital demands, it meets prespecified criteria for severity, persistence, perceived threat or functional impact.
Digital competence	Context-sensitive application of digital literacy, critical judgment and practical skills for effective and responsible digital participation.	Individual and context-dependent capacity.	Appraisal, decision-making, response-selection and applied digital tasks.	Broader applied capacity than digital literacy, but distinct from adversity-activated digital resilience.
Digital literacy	Foundational knowledge and skills for accessing, understanding, evaluating, creating and safely using digital information and technologies.	Individual knowledge and skills	Knowledge tests, risk-recognition tasks, critical evaluation of content.	A foundational component of digital competence; it does not require adversity or post-adversity recovery.
Digital resilience	Domain-specific manifestation of the broader resilience process, involving appraisal, coping, response regulation, and recovery under digital adversity.	Event, within-person and individual trajectory	Coping following online adversity, recovery latency, maintenance or restoration of functioning	Requires demonstrated digital adversity; positive or skilled digital use alone is insufficient.
Domain-level resources and vulnerabilities	Antecedent child or environmental characteristics that increase or reduce the likelihood of adaptive responses.	Individual, family, peer, school or technological context	Behavioral rhythms, family support, classroom climate, platform conditions	They precede or modify the resilience process but are not themselves resilience outcomes.
Effortful control	Temperament-related disposition towards voluntary attentional control and inhibition or activation of behavior.	Relatively stable individual difference	Parent- or child-reported attentional, inhibitory and activational control	Temperamental disposition rather than a synonym for task-based executive functioning or the complete self-regulation system.
Emotion regulation	Specific process through which emotional responses are identified as requiring regulation and strategies are selected and implemented.	Momentary, event or individual level	Reappraisal, attentional deployment, response modulation, EMA coping measures	Concerns emotional responses specifically and constitutes one component of broader self-regulation.
Executive functions	Cognitive control capacities supporting goal-directed behavior, particularly inhibitory control, working memory and cognitive flexibility.	Individual capacity and task performance	Inhibition, working-memory and shifting tasks	Cognitive capacity supporting self-regulation; it does not include the full emotional, behavioral or contextual regulation process.
Multidimensional wellbeing	Broader developmental outcome reflecting emotional, psychological, social and potentially physical functioning over time.	Longer-term outcome	WHO-5 or multidimensional wellbeing indicators	May be supported by resilience but can also be present without adversity; therefore, it is not equivalent to resilience.
Resilience process	Higher-order temporal process through which adaptive functioning is maintained, restored or reorganized under adversity.	Within-person trajectory across events or time	Reactivity and recovery trajectories, maintenance of functioning, adaptive reorganization	Inferred from functioning over time under adversity; not interchangeable with a trait, capacity or single coping response.
Self-regulatory capacity	Broad capacity to modulate cognition, emotion, behavior and physiological responses in relation to goals and demands.	Individual and within-person state.	Composite self-regulation measures, repeated regulatory-state assessments.	Broader than emotion regulation or executive functioning; it is an antecedent capacity rather than the resilience process itself.

Because several constructs operate across multiple ReDiKid domains and cannot be assigned exclusively to a single domain, the constructs are presented in alphabetical order to facilitate orientation and comparison.

This hierarchy does not imply that constructs operate only at one level or in one analytic role. Their role should nevertheless be specified a priori in each empirical model. For example, self-regulatory capacity may serve as an antecedent predictor, emotion regulation as an event-level mechanism, adaptive functioning as a proximal outcome, and wellbeing as a longer-term developmental outcome. This distinction prevents the same construct from being simultaneously defined as a predictor, mechanism, and outcome within a single model.

### 3.6 For Whom and in What context?

ReDiKid is intended primarily as a framework for examining resilience processes during late childhood in contexts where digital technologies form a meaningful part of children’s everyday lives. It does not assume that children have equivalent access to devices, connectivity, digital skills, supportive adults, inclusive schools, or safe and developmentally appropriate platforms. The relevance and strength of particular domains and pathways will therefore vary across cultural, socioeconomic, institutional, technological, and family contexts (Stoilova et al., 2021; UNICEF Innocenti, 2025).

The framework’s propositions should not be interpreted as universally invariant. For example, the protective role of parental mediation may depend on caregivers’ digital knowledge, available time, language, and access to support; school-based buffering may depend on institutional resources and inclusion; and digital competence may have different implications in contexts characterized by limited connectivity, restricted access, or different cultural norms concerning autonomy, communication, and family regulation. Similarly, the forms, meanings, and thresholds of digital adversity may vary across social and cultural settings (Ungar and Theron, 2020; Stoilova et al., 2021).

Children who are neurodivergent, disabled, affected by migration or displacement, members of linguistic or cultural minorities, or living in socioeconomically disadvantaged circumstances should not be conceptualized as inherently deficient or uniformly vulnerable. Their digital experiences may involve distinctive configurations of barriers, risks, resources, and opportunities. Digital environments may intensify exclusion or discrimination, but they may also support communication, identity, belonging, learning, and access to communities and services. Empirical applications should therefore examine relevant structural and identity-related characteristics as potential modifiers of exposure, pathway operation, and outcomes rather than treating them solely as control variables (Hassrick et al., 2021; UNICEF Innocenti, 2025).

Accordingly, studies applying ReDiKid should specify their cultural and institutional setting, patterns of digital access, inclusion and accessibility conditions, and the populations represented. Measures, adversity thresholds, and interpretations of adaptive functioning should be developmentally and culturally examined rather than assumed to transfer unchanged across populations. Evidence that a proposed pathway differs across contexts should be used to refine its scope conditions rather than interpreted automatically as a failure of the broader developmental-systems approach (Ungar and Theron, 2020).

## 4 Dynamic pathways of resilience

The ReDiKid framework proposes that resilience emerges through multiple interacting pathways across the psychophysiological, behavioral, contextual, and digital domains. These pathways describe theoretically grounded cross-domain processes through which regulatory capacities, behavioral routines, contextual resources, and digital experiences may jointly shape adaptive functioning. This perspective is consistent with resilience theory, which conceptualizes resilience as a dynamic process emerging from interactions between individuals and their environments rather than as a fixed trait (Masten and Barnes, 2018; ahmed Shafi et al., 2020).

The four pathways are proposed process hypotheses rather than established causal mechanisms. Their expected operation is specified below through directional propositions addressing within-person, between-person, and contextual processes. The propositions are not assumed to hold universally; however, they generate explicit predictions concerning temporal ordering, direction of association, and moderating conditions. Findings that diverge from these predictions may challenge, delimit, or motivate refinement of the corresponding proposition, particularly when observed consistently under the specified adversity, developmental, and contextual conditions.

### 4.1 Core pathways

Four core cross-domain pathways are proposed:

#### 4.1.1 Self-regulatory capacity pathway

Self-regulatory capacity refers to the antecedent cognitive, emotional, behavioral, and physiological capacities through which children modulate their responses to everyday demands. It is conceptually distinct from resilience: self-regulatory capacity represents a potential predictor or mechanism, whereas resilience is inferred from the temporal pattern of maintained or restored adaptive functioning under adversity.

Executive functions, emotion-regulation processes, and physiological regulatory flexibility may shape the magnitude of children’s responses to academic challenges, peer interactions, and digital adversity, as well as the speed and completeness of subsequent recovery. During late childhood, increasing integration between executive control and stress-regulatory systems may strengthen these capacities (Gunnar and Quevedo, 2007; Blair and Raver, 2015; Hutton et al., 2024).

Within digitally mediated environments, this pathway becomes particularly salient because visible social feedback, online comparison, and persistent accessibility may increase regulatory demands (Nesi et al., 2018). The pathway therefore predicts differences in reactivity and recovery rather than treating self-regulation as a direct synonym for resilience.

#### 4.1.2 Movement–regulation pathway

Physical activity, sleep duration, timing, regularity, and recovery processes may support autonomic flexibility and stress recalibration, thereby strengthening regulatory capacity under cognitive and emotional load. Evidence from developmental psychobiology suggests that movement behaviors and sleep duration, timing, and regularity play an important role in shaping stress responsivity and executive functioning (Blair and Raver, 2015).

Daily behavioral patterns may contribute to the regulation of stress physiology and emotional functioning (Gunnar and Quevedo, 2007; Blair and Raver, 2015). Adequate sleep duration, consistent sleep–wake schedules, and balanced movement patterns may support autonomic flexibility, whereas insufficient, fragmented, or irregular routines may heighten physiological stress reactivity (Michels et al., 2013). During late childhood, increasing behavioral autonomy may stabilize or disrupt these regulatory cycles, making their developmental role particularly relevant (Best and Miller, 2010).

#### 4.1.3 Social buffering pathway

Supportive relationships mitigate stress reactivity and facilitate adaptive responses to both offline and digital challenges. Ecological resilience models highlight relational resources as central promotive and protective factors (Masten and Barnes, 2018).

As peer interaction extends into digitally mediated spaces, social buffering processes increasingly operate across hybrid offline–online ecologies. The availability of parental support, peer belonging, and positive classroom climates may buffer socially evaluative stressors amplified by digital communication (Nesi et al., 2018). Parental mediation and co-regulation further shape children’s interpretation of digital experiences and their capacity to recover from socially evaluative stress (Livingstone et al., 2017; Vissenberg et al., 2022). Teacher mediation and support may also contribute to these buffering processes, particularly when online experiences intersect with classroom dynamics, peer relations, or school wellbeing (Thornberg et al., 2024). Broader institutional actors, including media organizations, regulators, and civil society organizations, may indirectly support these buffering processes by shaping safer and more developmentally appropriate digital environments.

#### 4.1.4 Digital competence pathway

Digital literacy refers to the foundational knowledge and skills required to access, understand, evaluate, create, and use digital information and technologies. Within the ReDiKid framework, digital literacy is conceptualized as a component of the broader construct of digital competence rather than as a parallel or interchangeable construct. Digital competence refers to the context-sensitive application of digital knowledge, critical understanding, judgement, and practical skills to engage effectively, responsibly, and meaningfully with digital environments.

Digital competence may support children in recognizing digital risks, critically appraising content and interactions, understanding how digital environments are structured, and selecting context-appropriate responses. It is therefore conceptualized as an antecedent capacity that may support adaptive engagement across varying levels of digital demand. However, competent digital engagement in the absence of adversity should not be interpreted as digital resilience.

Digital resilience is conceptually distinct from both digital literacy and digital competence. It refers specifically to the appraisal, coping, response regulation, and recovery processes activated when a child encounters digital adversity (Vissenberg et al., 2022; Pan et al., 2025). Digital competence may increase the likelihood that a risk is recognized and an appropriate response is selected, whereas digital resilience is reflected in how the child manages the experience, mobilizes available resources, and maintains, restores, or adaptively reorganizes functioning following adversity.

The digital competence pathway also recognizes that children’s digital experiences unfold within information ecosystems shaped by platform architectures, algorithmic amplification, commercial interests, institutional rules, social norms, and unequal power relations. These conditions influence which content becomes visible, credible, and widely circulated and therefore shape the demands placed on children’s critical understanding, judgement, and decision-making.

Digital competence may function promotively by supporting effective and meaningful participation across everyday digital contexts. Under conditions of digital adversity, it may also operate as an antecedent protective resource by facilitating risk recognition, critical appraisal, help-seeking, and the selection of appropriate responses. Digital resilience, by contrast, becomes observable only when adversity is present and is reflected in adaptive coping, response regulation, and recovery.

During late childhood, emerging metacognitive and self-reflective capacities may shape how digital experiences are interpreted and integrated into broader self-regulatory processes. These capacities are socially and technologically embedded and develop through interactions with family, peers, schools, platforms, and broader institutional contexts (Hammond et al., 2024).

Digital competence and digital resilience should therefore demonstrate related but discriminant patterns of association. Digital competence is expected to be more strongly associated with risk recognition, critical appraisal, and response selection, whereas digital resilience is expected to be more strongly associated with coping, response regulation, and recovery following digital adversity. This distinction is formalized in Proposition 5 (Table 4).

**TABLE 4:** Empirically testable propositions derived from the ReDiKid framework.

Proposition	Primary level of analysis	Expected empirical pattern	Findings that would challenge or refine the proposition
P1. Adversity activation criterion and adaptive functioning. Resilience should be inferred only when a child has been exposed to an event or condition meeting the prespecified criteria for acute or chronic adversity and adaptive functioning is subsequently maintained, restored, or adaptively reorganized relative to an appropriate pre-adversity baseline or developmental expectation. In the absence of identifiable adversity, positive functioning should be interpreted as competence or wellbeing rather than as evidence of resilience.	Within-person or event level.	Following adversity, maintenance of adaptive functioning, a smaller deviation from the child’s baseline, or faster restoration or adaptive reorganization of functioning indicates a more favorable resilience process.	The proposition would require refinement if indicators interpreted as resilience demonstrated equivalent construct validity and predictive utility regardless of whether prespecified adversity was present, or if adversity-conditioned and non-adversity-conditioned adaptive functioning could not be meaningfully distinguished.
P2. Self-regulatory capacity pathway. After accounting for the severity of digital adversity, more favorable pre-event regulatory functioning will predict lower peak dysregulation and shorter recovery latency. Within-person analyses will examine fluctuations relative to the child’s typical regulatory state, while complementary between-person analyses may examine more stable individual differences in regulatory capacity.	Primarily within-person and event level; complementary between-person analyses may examine stable individual differences.	Higher pre-event regulatory capacity predicts lower emotional or physiological reactivity and faster recovery following digital adversity.	Pre-event regulatory capacity is unrelated to the magnitude or duration of post-event dysregulation, or associations consistently occur in the opposite direction under comparable conditions.
P3. Movement–regulation pathway. Within a child, less favorable 24-h movement patterns, such as shorter or more irregular sleep, lower physical activity, or disrupted behavioral rhythms, will predict greater subsequent emotional or physiological reactivity and slower recovery following digital adversity, after accounting for stable between-person differences.	Within-person, day-to-day level.	Shorter or more irregular sleep, lower physical activity, or greater disruption of 24-h movement-behavioral patterns predict higher subsequent emotional or physiological reactivity and longer recovery latency.	No temporally ordered within-person association is observed; the association occurs only between children; or less favorable movement patterns consistently predict more favorable recovery.
P4. Social-buffering pathway. At the event, day, or longer-term contextual level, the association between digital adversity and subsequent dysregulation or recovery latency will be weaker when children have greater access to context-relevant support from parents, peers, teachers, or other trusted adults.	Event, day-to-day, and longer-term contextual level.	Greater access to relevant social support attenuates the association between adversity severity and subsequent dysregulation or recovery latency and may additionally be associated with faster recovery.	Relevant and accessible social support does not moderate the association between adversity and subsequent functioning, or greater support consistently intensifies dysregulation under comparable conditions.
P5. Digital competence and digital resilience. Digital competence and digital resilience are expected to show related but discriminant patterns of association. Digital competence should be more strongly associated with risk recognition, critical appraisal, and response selection, whereas digital resilience should be more strongly associated with regulation, coping, and recovery following digital adversity.	Construct, event, and individual level.	Digital competence is expected to be more strongly associated with risk recognition, critical appraisal, and response selection, whereas digital resilience is expected to show stronger associations with coping, response regulation, and recovery following digital adversity.	Digital competence and digital resilience are empirically indistinguishable or demonstrate equivalent temporal and functional relationships with adversity appraisal and post-adversity recovery.
P6. Cross-domain configurations. The association between digital adversity and impaired adaptive functioning will be stronger when vulnerabilities across multiple domains co-occur. Conversely, combinations of regulatory capacity, stable behavioral routines, and relevant contextual support will provide incremental explanatory value for recovery beyond the independent contributions of individual domains.	Multilevel, incorporating individual, daily, event-level, and contextual characteristics.	Accumulated cross-domain vulnerabilities predict greater dysregulation and slower recovery, whereas combinations of cross-domain resources predict more favorable recovery beyond the independent contribution of individual resources.	Cross-domain configurations do not explain adaptive functioning or recovery beyond the independent contributions of individual domains, or the predicted cumulative pattern consistently operates in the opposite direction.

For clarity, the core characteristics of the proposed pathways and their roles within the ReDiKid framework are summarized in Table 5.

**TABLE 5:** Core pathways and their roles within the ReDiKid framework.

Pathway	Core function	Key domains involved	Promotive role	Protective role
Self-regulatory capacity	Cognitive, emotional, behavioral, and physiological capacities supporting the modulation of responses to daily demands.	Psychophysiological Behavioral Digital	Supports anticipatory and ongoing management of everyday demands.	Predicts lower reactivity and faster recovery under social and digital adversity.
Movement–regulation	Regulation through physical activity, sleep, and recovery processes.	Behavioral Psychophysiological	Supports physiological balance and cognitive functioning.	Reduces stress reactivity and fatigue.
Social buffering	Ability to form and maintain supportive relationships.	Contextual Digital	Enhances a sense of belonging, stability, and access to support.	Buffers socially evaluative, relational, and digitally mediated stress.
Digital competence	Context-sensitive application of digital knowledge, critical understanding, judgement, and practical skills.	Behavioral Contextual Digital	Enables effective, responsible, and meaningful digital participation.	Supports risk recognition, critical appraisal, help-seeking, and the selection of appropriate responses under digital adversity.

### 4.2 Empirically testable propositions

The ReDiKid framework generates an initial set of directional propositions that can be examined through study-specific empirical models. The propositions distinguish adversity exposure, regulatory mechanisms, and adaptive outcomes and specify the level of analysis, expected empirical pattern, and findings that would challenge or motivate refinement of each proposition. They are intended to be evaluated and progressively refined through cumulative research, with converging evidence potentially supporting, delimiting, or challenging individual propositions (Table 4).

Not every empirical study is expected to test all six propositions, nor is each ReDiKid study expected to support the proposition it examines. Each study should identify a priori which proposition or propositions are being addressed and specify the relevant operational definitions, temporal ordering, directional predictions, and scope conditions. Findings that diverge from the predicted pattern should be used to evaluate and, where appropriate, refine the proposition’s direction, boundary conditions, temporal assumptions, or level of analysis. Consistent or accumulating evidence that challenges a proposition may ultimately lead to its restriction or rejection rather than being retrospectively accommodated within the framework. Interpretation of non-support should consider the adequacy of construct operationalisation, measurement validity, statistical power, temporal resolution, and the prespecified scope conditions of the proposition. These considerations should be specified a priori where possible and should not be used retrospectively to accommodate any empirical outcome.

### 4.3 Methodological architecture for dynamic pathway analysis

Methodological orientation is consistent with approaches that conceptualize resilience as a dynamic and temporally embedded process (Shiffman et al., 2008; ahmed Shafi et al., 2020; Masten, 2021), requiring methods capable of capturing intra-individual variability and context-sensitive change in naturalistic settings. To empirically operationalise the dynamic pathways described above, the ReDiKid framework supports the use of intensive longitudinal and multimodal research designs that complement traditional cross-sectional approaches. Because resilience is conceptualized as a temporally embedded, cross-domain, and developmentally evolving process, its investigation requires methodologies capable of capturing within-person dynamics, cross-domain coupling, and developmental recalibration of regulatory systems across late childhood.

#### 4.3 1 Methodological approaches

The ReDiKid framework supports the use of complementary methodological approaches selected according to the specific research question and the temporal and contextual scale of the processes under investigation. These approaches include longitudinal cohort designs, intensive longitudinal and ambulatory assessment, laboratory-based studies, and qualitative or mixed-methods research.

Intensive longitudinal methods, particularly ecological momentary assessment (EMA), are well-suited to capturing short-term within-person variability in emotional states, regulatory responses, contextual exposures, and digital engagement within children’s natural environments. When combined with wearable sensors or passive digital tracking, these methods may enable the examination of temporal coupling across psychophysiological, behavioral, contextual, and digital processes.

Qualitative and interpretive approaches, including in-depth interviews, focus groups, and diary-based methods, may complement quantitative designs by capturing lived experiences, meaning-making processes, and situated interpretations of digital environments. They may also provide insight into how social norms, institutional arrangements, narratives, and power relations shape children’s experiences and responses. Such insights may help translate pathway-based findings into context-sensitive interventions and recommendations for educational, institutional, and policy settings.

Longitudinal designs are particularly relevant for examining developmental trajectories and changes in pathway strength, cross-domain coupling, and regulatory processes across late childhood and the transition into adolescence. Employing all of these approaches in a single study may be neither feasible nor practical; rather, the framework provides a methodological architecture within which different designs can examine selected components and pathways. Across these methodological approaches, different forms of self-reported, caregiver-reported, sensor-derived, and digitally recorded data may be combined to examine cross-domain processes at complementary temporal and contextual scales.

#### 4.3.2 Integration of multiple data sources

Building on this methodological diversity, the ReDiKid framework supports the integration of multiple data sources to capture psychophysiological, behavioral, contextual, and digital processes at relevant temporal scales. Individual studies may combine different forms of data depending on the research question and design. Self-reported data may include repeated assessments of emotional states, perceived stress, coping strategies, social context, and the purpose of digital engagement, collected using EMA protocols and validated questionnaires. In parallel with child assessments, longitudinal parent-reported data collected within the same measurement waves will enable the construction of a parent–child data structure, allowing the modeling of family-level digital cultures, parental monitoring practices, and regulatory scaffolding.

To complement these subjective and caregiver-reported measures, sensor-derived and digitally recorded data may provide time-stamped indicators of behavioral, physiological, digital, and environmental processes. These data may include:

1. wearable accelerometry providing estimates of daily movement patterns, including sleep duration and regularity, physical activity, and sedentary behavior;
2. continuous heart-rate monitoring providing indicators of autonomic regulation and recovery, such as heart-rate variability;
3. passive digital tracking providing time-stamped records of device use, application patterns, and the temporal fragmentation of digital engagement;
4. where relevant, sensor-based indicators of environmental exposures, such as ambient light, noise, temperature, or air quality, which may shape sleep, stress regulation, and daily functioning.

Where appropriate, data streams may be synchronized and time-stamped, enabling the alignment of physiological signals, behavioral rhythms, contextual conditions, and digital exposures. This integration of multiple data sources may facilitate high-resolution modeling of cross-domain dynamics within daily cycles.

Sensor-derived and digitally recorded indicators should be interpreted in conjunction with self-reported, caregiver-reported, and contextual information, acknowledging that their meaning depends on measurement procedures, analytical decisions, and relational and situational contexts.

#### 4.3.3 Analytical modeling

Building on the integration of multiple data sources, the ReDiKid framework supports the use of analytical methods selected according to the research question, study design, temporal resolution, and structure of the available data. Depending on these characteristics, analyses may examine within- and between-person variability, developmental change, cross-domain associations, and reciprocal or non-linear processes.

Theory-informed statistical modeling techniques, such as multilevel modeling and structural equation modeling, may be used for testing hypothesized cross-domain pathways and examining mediation, moderation, and buffering mechanisms. Where appropriate, complementary analytical methods may be used to address specific forms of complexity. For example, person-oriented and latent-profile methods may help identify heterogeneous resilience profiles, while Bayesian modeling or machine-learning techniques may be useful for probabilistic estimation, prediction, or the exploration of complex interactions. Their use is not required by the framework and should be determined by the research question, sample size, measurement design, and analytical purpose.

Dynamic network analysis may provide an additional strategy for modeling temporally ordered and potentially reciprocal associations among psychophysiological, behavioral, contextual, and digital domains, enabling identification of central nodes and leverage points within the resilience system.

Across these analytical options, data-driven methods should complement rather than replace theory-informed inference. Their contribution may include detecting regulatory patterns, identifying heterogeneous resilience profiles, testing hypothesized pathways, and generating findings that can inform preventive, educational, and policy-oriented applications.

#### 4.3.4 Ethical and developmental safeguards

The intensive, data-rich research designs supported by the ReDiKid framework require a developmentally sensitive ethical framework. Research involving wearable sensors, passive digital tracking, and ecological momentary assessment requires careful attention to privacy, confidentiality, autonomy, and psychological safety.

Data governance procedures should prioritize minimisation, pseudonymisation, and secure storage of high-resolution behavioral and physiological data. Age-appropriate consent and assent procedures should be implemented in age-appropriate formats and through an ongoing process of information and agreement, supporting children’s emerging autonomy while maintaining appropriate parental oversight.

Continuous digital and physiological monitoring should be transparent, voluntary, and interruptible. Ethical safeguards are treated as integral components of the developmental systems approach, recognizing that the research process itself forms part of the child’s ecological environment.

## 5 ReDiKid as a research programme

ReDiKid is not a protocol for a single intervention, cohort, or data-collection study and therefore does not prescribe a fixed schedule for participant recruitment, intervention delivery, or assessment. Instead, it provides a common conceptual foundation for complementary, study-specific protocols. Each protocol specifies its own research design, target population, recruitment strategy, eligibility criteria, timeline, and measurement procedures, while operationalising selected components and pathways of the shared ReDiKid framework.

ReDiKid is conceived as a research programme that progresses from population-level mapping toward increasingly fine-grained analyses of children’s everyday regulation within digital ecologies. The framework establishes a coordinated architecture within which complementary studies examine distinct yet interconnected components of child resilience.

Across this coordinated programme, individual studies may aim to:

1. empirically examine a multidimensional, cross-domain model of child resilience in digital environments;
2. systematically test hypothesized regulatory pathways linking digital engagement, time use, movement behaviors, and psychophysiological functioning;
3. integrate data from multiple sources across laboratory, ambulatory, and real-world settings, combining sensor-based indicators with intensive longitudinal self-report data; and
4. translate theoretical and empirical insights into evidence-informed recommendations for education, mental-health promotion, prevention of digital harms, and policy.

Individual studies are not expected to operationalise all components and pathways of the framework simultaneously. Instead, each investigation can examine selected mechanisms within a shared conceptual structure, allowing cumulative theory refinement across diverse methodological designs. This staged architecture reflects a developmental systems logic in which understanding emerges through coordinated empirical studies conducted across successive stages of the research programme.

## 6 Implications for research, education, prevention, and policy

As a conceptual framework, ReDiKid provides a shared structure for integrating psychophysiological, behavioral, contextual, and digital perspectives across complementary studies. It guides the selection of research questions, measures, and analytical strategies, while allowing individual studies to focus on selected domains and pathways. Future research may further examine how emerging digital conditions, including platform architectures, algorithmic content curation, persuasive design, and artificial intelligence, shape children’s regulatory demands and resilience processes. These directions represent areas for future theoretical and empirical refinement rather than additional structural pathways. ReDiKid may also be considered alongside models of compensatory and problematic digital engagement, including the Compensatory Internet Use Model (Kardefelt-Winther, 2014) and the Interaction of Person–Affect–Cognition–Execution model (I-PACE; Brand et al., 2016, 2019, 2025), to examine when digital engagement serves adaptive or compensatory regulatory functions and when it develops into a persistent, inflexible, or functionally impairing pattern.

In educational and preventive contexts, the framework highlights the importance of combining individual skill development with supportive environments. Relevant strategies may include social and emotional learning, media and digital literacy, supportive peer and teacher relationships, opportunities for movement and recovery across the school day, and targeted support for children experiencing online adversity. Physical education may contribute to this broader whole-school approach by supporting movement competence, mastery experiences, social participation, and behavioral regulation, rather than functioning as a stand-alone intervention.

From a public-health and policy perspective, ReDiKid supports integrated approaches linking mental wellbeing, physical activity, sedentary behavior, sleep, social support, and digital experiences. Evidence generated within the framework may inform education policy, child online-safety and platform-governance policy, public-health and prevention strategies, family and community support, and research and data-governance standards. Such policies should address the institutional, commercial, social, and technological conditions that shape children’s digital experiences (OECD, 2019; Ministerstvo zdravotnictví Èeské republiky, 2020; WHO Regional Office for Europe, 2025). The policy examples discussed in this article are drawn primarily from European and other high-income settings and should not be interpreted as globally representative. Future applications of ReDiKid should incorporate evidence and perspectives from low- and middle-income countries and from culturally, linguistically, socioeconomically, and developmentally diverse populations. Policy implications should be adapted to local patterns of connectivity, digital access, institutional capacity, children’s rights, and available family and community support (Stoilova et al., 2021; UNICEF Innocenti, 2025).

### 6.1 Limitations and directions for future research

As a conceptual framework, ReDiKid has several limitations that should be addressed through future empirical research. First, the proposed domains, pathways, and propositions have not yet been empirically validated as an integrated system. Their evaluation will depend on the validity and developmental appropriateness of measures used to operationalise adversity, regulatory capacities, digital competence and digital resilience, adaptive functioning, and recovery. Some constructs may also show empirical overlap, and their operationalisation may differ across event-, day-, and developmental timescales. Second, causal inference remains challenging because the proposed processes are reciprocal, time-varying, and incorporated into family, school, and digital contexts. Longitudinal and intensive within-person designs may strengthen temporal inference but cannot, by themselves, establish causality; where ethically and practically feasible, quasi-experimental, natural-experimental, and intervention designs may provide complementary evidence. Finally, the generalisability of ReDiKid remains to be established. The relevance, operationalisation, and relative strength of its pathways may vary across developmental stages, cultural and socioeconomic contexts, disability and neurodivergence, and patterns of digital access. Future research should therefore examine measurement equivalence, contextual moderators, and boundary conditions across diverse populations and use cumulative evidence to refine, restrict, or, where appropriate, reject individual propositions.

## 7 Conclusion

ReDiKid introduces a transdisciplinary conceptual framework for understanding child resilience in the digital age. By integrating developmental systems theory with digital ecology and a temporal behavior perspective, the framework supports a shift from static risk mapping toward dynamic modeling of adaptive capacity in digitally embedded childhood.

This paper establishes the theoretical foundation for a coordinated programme of empirical studies and translational applications.

By conceptualizing resilience as a temporally embedded, system-level process emerging from dynamic interactions across psychophysiological, behavioral, contextual, and digital domains, ReDiKid shifts attention from risk exposure alone toward adaptive capacity building across interconnected systems. In doing so, the framework provides a foundation for further research on child development in digitally mediated environments and for evidence-informed strategies that support children not only in navigating digital environments safely, but also in developing strengths, resources, and adaptive capacities within them.

## Data Availability

The original contributions presented in this study are included in this article/supplementary material, further inquiries can be directed to the corresponding author.
